# Extreme events, trophic chain reactions, and shifts in phenotypic selection

**DOI:** 10.1038/s41598-023-41940-6

**Published:** 2023-09-13

**Authors:** Kate Layton-Matthews, Stefan J. G. Vriend, Vidar Grøtan, Maarten J. J. E. Loonen, Bernt-Erik Sæther, Eva Fuglei, Brage Bremset Hansen

**Affiliations:** 1 Department of Biology, Centre for Biodiversity Dynamics, NTNU, Trondheim, Norway; 2 Norwegian Institute for Nature Research, NINA, Tromsø, Norway; 3 Department of Animal Ecology, Netherlands Institute of Ecology (NIOO‐KNAW), Wageningen, The Netherlands; 4 Arctic Centre, University of Groningen, Groningen, The Netherlands; 5 Norwegian Polar Institute, Tromsø, Norway; 6 Department of Terrestrial Ecology, Norwegian Institute for Nature Research, NINA, Trondheim, Norway

**Keywords:** Climate-change ecology, Community ecology, Ecological modelling, Population dynamics

## Abstract

Demographic consequences of rapid environmental change and extreme climatic events (ECEs) can cascade across trophic levels with evolutionary implications that have rarely been explored. Here, we show how an ECE in high Arctic Svalbard triggered a trophic chain reaction, directly or indirectly affecting the demography of both overwintering and migratory vertebrates, ultimately inducing a shift in density-dependent phenotypic selection in migratory geese. A record-breaking rain-on-snow event and ice-locked pastures led to reindeer mass starvation and a population crash, followed by a period of low mortality and population recovery. This caused lagged, long-lasting reductions in reindeer carrion numbers and resultant low abundances of Arctic foxes, a scavenger on reindeer and predator of migratory birds. The associated decrease in Arctic fox predation of goose offspring allowed for a rapid increase in barnacle goose densities. As expected according to *r*- and *K*-selection theory, the goose body condition (affecting reproduction and post-fledging survival) maximising Malthusian fitness increased with this shift in population density. Thus, the winter ECE acting on reindeer and their scavenger, the Arctic fox, indirectly selected for higher body condition in migratory geese. This high Arctic study provides rare empirical evidence of links between ECEs, community dynamics and evolution, with implications for our understanding of indirect eco-evolutionary impacts of global change.

## Introduction

Global change impacts biota through a range of direct and indirect effects on eco-evolutionary processes^1–3^. For instance, by changing the density of a keystone species, environmental change can indirectly alter other species’ densities and their community-level dynamics, and even lead to trophic cascades^4,5^, operating through species interactions^6,7^. Phenotypic selection can alter densities and affect population dynamics when acting on density-regulated vital rates^8,9^. Changing densities may, in turn, influence the relative fitness of traits^10^, inducing trade-offs between trait values that maximise fitness at different population sizes^11^. In spite of increased research interest on the implications of density-dependent selection and several experimental studies supporting density dependence as a selective agent [e.g.,^8,12–14^], empirical evidence from the wild remains elusive^9^. Eco-evolutionary feedbacks can however be expected between traits and demography, caused by shifts in selective pressure associated with environmental changes^15,16^. By extension, environmentally-induced changes in species’ abundances, and/or in their trophic interactions, have the potential to shape selection on phenotypic traits^17^. In the long run, this can result in co-adaptation of interacting species^9^. In this way, sudden environmental change could have cascading evolutionary consequences, especially when the effects are long-lasting^8,17,18^. This is particularly relevant in the context of global warming, which is causing more, and stronger, environmental perturbations due to increasingly frequent and intense extreme climatic events [ECEs,^19^]. ECEs are, per definition, rare. Thus, despite growing evidence of their ecological consequences^19,20^, empirical studies of any short- or long-term eco-evolutionary effects of ECEs have generally proven difficult^21^.

In this study, we provide rare empirical evidence of density-dependent phenotypic selection in the wild, enabling us to link a sudden, lasting change in phenotypic selection regime to a trophic chain reaction triggered by an ECE. Here, we refer to a ‘trophic chain reaction’ rather than ‘tropic cascade’ [sensu^22^], which refers strictly to downward propagating effects of predators, involving three or more trophic levels. We used time series data of rain-on-snow events, wild Svalbard reindeer (*R*. *t*. *platyrhynchus*) abundance, a proxy of Arctic fox (*Vulpes lagopus*) abundance, and individual-based and abundance data from a population of breeding barnacle geese (*Branta leucopsis*) on high Arctic Svalbard. Such high-latitude Arctic environments are seasonal and highly stochastic, and their tundra vertebrate communities are characterised by relatively few species and trophic levels, yet strong trophic interactions i.e., strong effects of a change in one species’ abundance on another interacting species^23,24^. Because environmental effects on population dynamics are typically also strong [e.g.,^24^], these interactions may be particularly sensitive to ongoing rapid climate change. For instance, resident herbivores (notably reindeer and caribou) are now increasingly often subject to ‘ice-locked’ winter pastures due to extreme warm spells and rain-on-snow events^25^, which can cause mass starvation episodes, with potential knock-on effects on interacting species^24^. Following density-dependent selection theory^26^, we tested for change in goose phenotypic selection pressure on a key morphological trait (body condition) in response to the changes in species’ abundances associated with this ECE. Specifically, we tested whether resultant higher population densities of geese, due to reduced predation of their young by Arctic foxes, caused selection for higher body condition.

## Results and discussion

**Figure 1:**
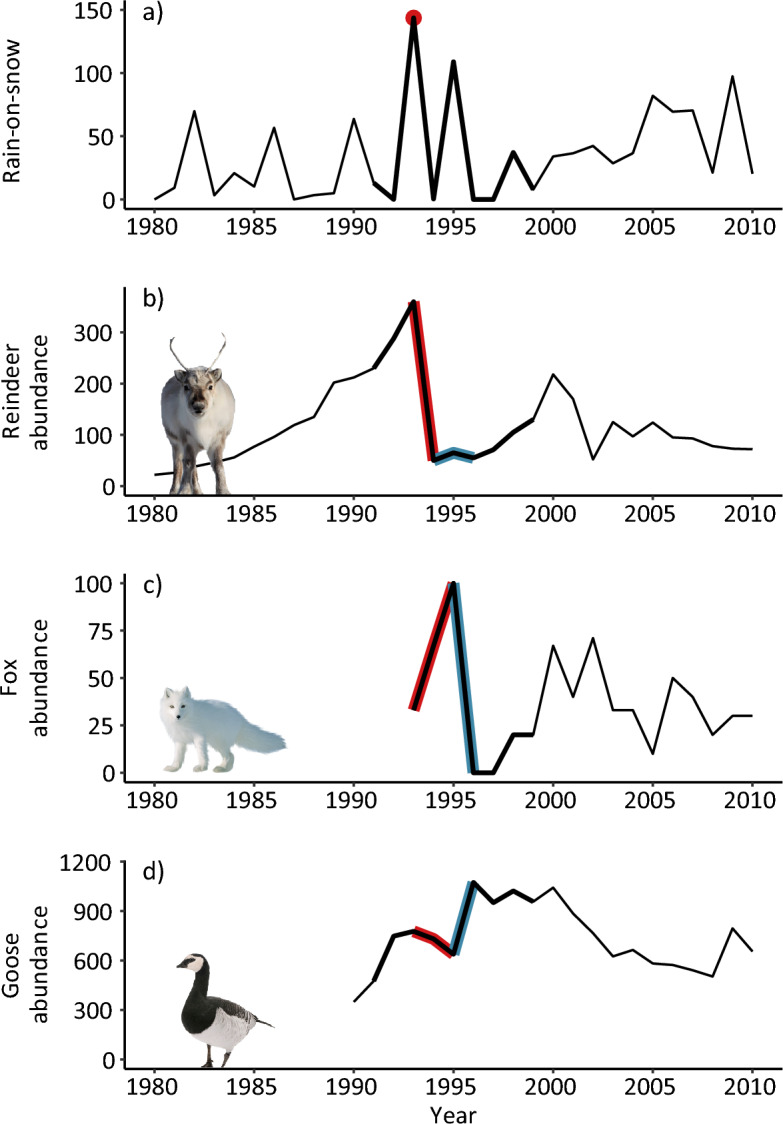
Time series of (**a**) rain-on-snow (total winter rainfall in mm in Ny-Ålesund, 1980–2010), (**b**) reindeer abundance (1980–2010), (**c**) Arctic fox abundance proxy (percentage occupied dens, 1993–2010) and (**d**) barnacle goose total population size (1990–2010). Coloured lines represent direct and lagged consequences of the rain-on-snow event in winter 1993–94 (red point in **a**). The event caused a population crash (**b**, red line) and a peak in reindeer mortality/carcasses that winter, then high density-dependent survival in subsequent years (b, blue line). This in turn caused high (**c**, red line) and then reduced (**c**, blue line) fox abundance, ultimately leading to supressed (**d**, red line) and then persistently higher barnacle goose reproduction and abundance (**d**, blue line). The thicker black lines represent the study period over which density-dependent selection was analysed (1991–1999).

**Figure 2:**
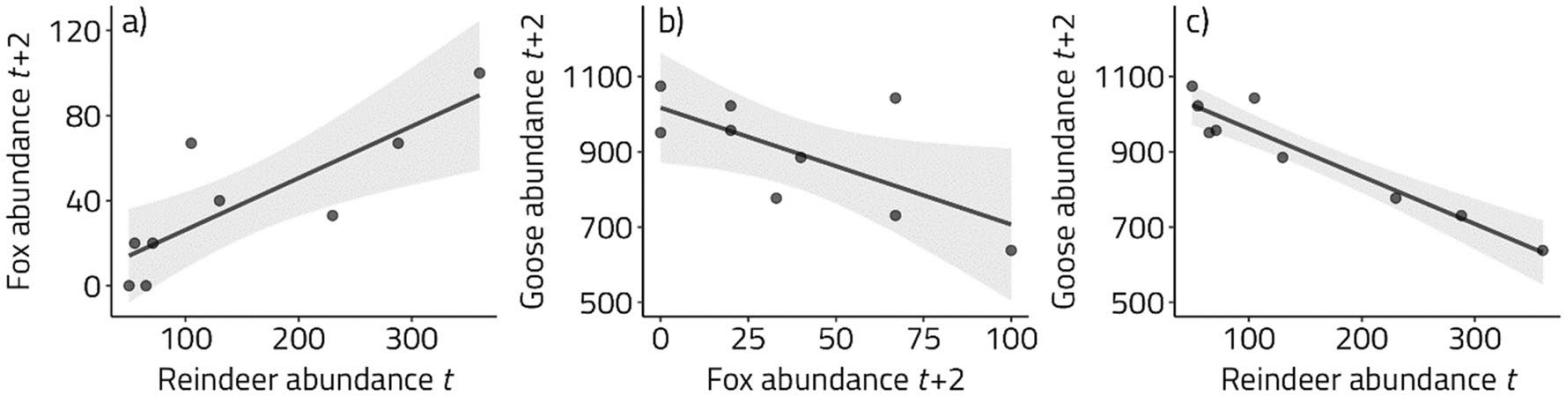
Pairwise correlations between (**a**) reindeer abundance (*t*) and fox reproduction (*t* + 2), (**b**) fox reproduction (*t* + 2) and total goose abundance (*t* + 2) and (**c**) reindeer abundance (*t*) and fox reproduction (t + 2) for the study period 1991–1999, shown as slope estimates with 95% confidence intervals, where points represent the raw data values.

In the winter of 1993–94, a winter ECE with record-breaking levels of rain and resultant ‘ice-locked’ winter pastures occurred in Svalbard^27,28^. This event led to a mass die-off in a wild Svalbard reindeer population close to Ny-Ålesund, Svalbard^29^ (Fig. 1b). Prior to this population crash, carcasses from this irrupting, and increasingly overabundant, reindeer population had already become a large and important food source for its scavenger, the Arctic fox. The reindeer population crash caused a ‘pulse’ of excessive food (i.e., carcass) availability that further boosted fox reproduction for two years. This led to high predation rates on the offspring of migratory barnacle goose^28,30^. Importantly, subsequent low reindeer numbers and mortality rates (via density dependence)^31^, and a corresponding long-lasting lack of carcasses for Arctic foxes, led to a substantial, and persistent, reduction in fox abundance^24,28^. This apparently allowed for high goose reproduction and increased goose densities in the years following the ECE^28,30,32^. Accordingly, this trophic chain reaction, with a strong positive correlation between reindeer abundance in year *t* and fox reproduction in year *t* + 2 (Pearson’s correlation coefficient = 0.83 [95% CIs 0.36,0.96], Fig. 2a) and a negative correlation between fox reproduction (*t* + 2) and goose abundance (*t* + 2) (− 0.69 [− 0.92, − 0.05], Fig. 2b), can explain the strong negative relationship between reindeer abundance in year *t* and goose abundance in year *t* + 2 (− 0.95 [− 0.99, − 0.79]) during our study period (Fig. 2c).

Thus, a single extreme climatic (rain-on-snow) event^31^ led to a lagged chain reaction by causing a peak in reindeer mortality (between 1993 and 1994), first increasing (1994–1995) and subsequently reducing (1996 and beyond) fox abundance, and ultimately leading to a shift in goose production and abundance (1996 and beyond) (Fig. 1).

**Figure 3:**
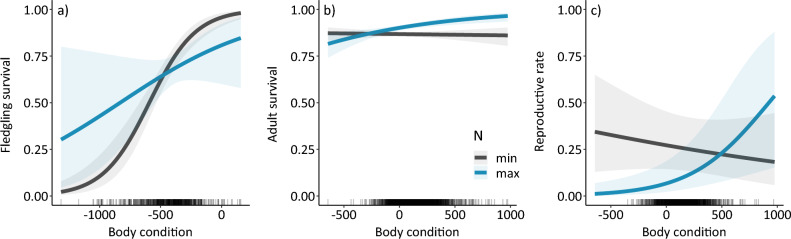
Model predictions with 95% confidence intervals of the effect of barnacle goose body condition on (**a**) fledgling survival, (**b**) adult survival and (**c**) the reproductive rate, at the minimum (N = 566, black line) and maximum (N = 1071, blue line) population size. Data distributions are shown on the x‐axis as rugs.

Body condition (body mass adjusted for structural size, in our case tarsus length) is highly dynamic in geese, reflecting fluctuations in food and habitat resources^33^. A change in phenotypic selection pressure through body condition assumes an association between the phenotypic trait and demographic rates that underly population abundances. Such associations can also be density dependent [e.g.,^34^]. Accordingly, we found a positive relationship between body condition and post-fledging survival of barnacle geese, where the effect was strongest at low densities (Fig. 3a). Survival of first-year (post-fledging) birds is generally lower and more variable than adult survival. Additionally, body condition has been shown to strongly affect fledgling survival^35,36^. For adults, there was a positive effect of body condition on their survival (Fig. 3b) and reproductive rates (probability of a female adult producing a fledgling, Fig. 3c) at high densities but a negligible effect at lower densities. As capital breeders, accumulation of fat reserves, i.e., improved body condition, is important for female barnacle geese prior to reproduction^37,38^. When fewer resources are available, this reduces individuals’ reproductive output overall but also increases intraspecific competition, meaning that individuals of poorer quality can be disproportionately impacted^39^.

There was little support for a density-dependent effect of body condition on fledged brood sizes (Appendix S1 Table S1.1.3). Furthermore, results from the model selection with body mass as response were the same as those for body condition (Appendix S1 Table S1.2.3). Time series data from 1991 to 2017 were used to estimate demography-trait relationships. Analyses were also run using only the same years as for the selection analyses (i.e., with parentage data, 1991–1999). In this case with a much shorter study period, simpler models with additive effects of mass and density (no interactions) were the best-fitting models of survival and reproductive rates.

**Figure 4:**
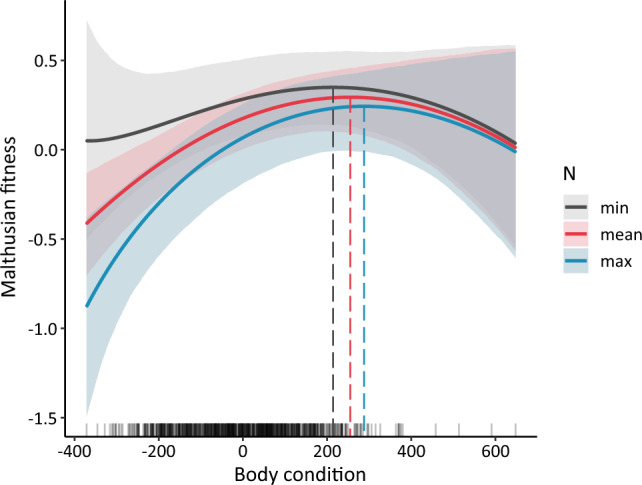
Malthusian fitness (Eq. 2) for values of barnacle goose body condition at minimum (N = 566), mean (800) and maximum (1071) population size, N. Thick lines represent posterior means. Ribbons represent 95% credible intervals. Dashed lines refer to body condition values maximising fitness.

Barnacle goose population size (N) has changed markedly over the study period (1991–1999), with a marked shift in 1996 (Fig. 1d). The smallest estimated annual population size was 566 and the largest was 1071. Population growth is density regulated primarily through reproduction^40^, and largely due to competition for resources during the breeding season^30^. As expected from density-dependent selection theory, increasing population sizes were associated with a decline in Malthusian fitness (Fig. 4). The goose body condition maximising fitness was higher with increasing population density (Fig. 4), i.e., the phenotype maximising fitness differed with proximity to the carrying capacity. This density-phenotype interaction was significant for body condition and body mass but not for tarsus length (Appendix S2 Table S2.1–S2.3 and Fig. S2.1–S2.2), indicating that condition, rather than size, is under density-dependent selection.

Such density-dependent selection on fitness-related traits can be expected due to observed resource competition at the breeding grounds. Competition for nesting sites^41^ and food^42,43^ are likely the main mechanisms behind density-dependent reproduction in barnacle geese^40^. Furthermore, individuals in better condition are more dominant, gaining access to higher quality resources^38^, and such competitive skills appear increasingly favourable at higher densities. Thus, larger population sizes mean greater resource competition and stronger selection for improved body condition.

Predation can be a strong agent of density regulation in their prey [e.g.,^44^], even leading to trophic cascades [e.g.,^45^]. Arctic fox predation impacts barnacle goose population sizes through effects on gosling survival^28,30,32^. Thus, an initial increase—followed by a persistent drop—in Arctic fox reproduction, and hence abundance^24^, associated with the ECE-induced chain reaction (Fig. 1c), altered the selection pressure on goose body condition. During the first part of the study period (until 1995), with goose population densities supressed by increasingly high fox predation pressure^28^, the body condition maximising Malthusian fitness was under stabilising selection (b_3_ = – 0.044 (median), − 0.084–− 0.006 (95% credible interval, Appendix S2 Table S2.1). In the second period (1996–1999), with lower fox abundance and resultant higher goose densities, body condition appeared to be under positive, directional selection (b_2_ = 0.207, 0.138–0.283, Appendix S2 Table S2.1). This provides rare evidence from the wild of selection favouring individuals with better competitive skills (higher body condition) at higher densities, i.e., density-dependent selection. Note, however, that these results are strictly correlative and we cannot exclude other—alternative or complementary—explanations for this shift in selection regime, such as non-lethal predator effects^46^ associated with changes in optimal body condition, e.g., avoidance behaviour^47^.

Such feedbacks between the ecological effects of intra-specific competition and the selective response (i.e., shift in optimal body condition) have eco-evolutionary implications. In barnacle geese, both body mass and body size are heritable, in other words they can respond evolutionarily to phenotypic selection^48,49^. While the longer-term evolutionary consequences of ECEs for wild populations remain unclear, there is some evidence for selective responses to sudden, intense perturbations^50^. Although most field studies have focused on the evolutionary responses of gradual, sustained environmental change^21^, the selective pressures imposed by ECEs may still represent a major driver of evolutionary change^51,52^. For example, a severe cold spell selected for larger body size in a cliff swallow (*Petrochelidon pyrrhonota*) population, i.e., favouring individuals better able to survive extreme conditions^53^. Similarly, extreme drought favoured larger individuals in medium ground finches (*Geospiza fortis*)^54^. Here, in this high-arctic ecosystem^55^, we have documented how an extreme rain-on-snow event induced a chain reaction, where the lagged effects of this ECE ultimately increased the densities and, thereby, optimal body condition of a migratory Arctic herbivore. While there is growing evidence from the wild that environmental change affects trophic interactions and phenotypic dynamics^56–58^, to our knowledge this study is the first to provide evidence of changing selection caused by such a chain of trophic interactions. This fundamental eco-evolutionary feedback, in turn, has the potential to influence long-run trophic interactions and community dynamics^8^.

Fluctuations in the environment can affect which phenotypic trait values maximize relative fitness^26,59,60^. Despite our evidence of directional selection for higher body condition (and larger body mass) at high population densities, there was no significant trend in average body condition (*p*-value = 0.99) over the selection study period 1991–1999, despite a statistically significant decrease in tarsus length (*p*-value < 0.001, Appendix S3 Fig. S3.1). A study from this population, based on data from 1991 to 2017, showed that body condition has undergone a significant decline over this (longer) period, although the impact on population growth was minimal^61^. Declines in body condition, despite positive selection, have been also observed in several Arctic breeding goose species and attributed to long-term habitat degradation and increasingly limited food resources^36,49^.

Empirical documentation of links between ECEs and evolutionary change remains rare, despite their probable prevalence. We have demonstrated how effects of a single ECE in a rapidly changing high Arctic environment indirectly induced an evolutionary response in a migratory species not even present during the event. As such, ECEs are predicted to become more frequent under global warming, and their impacts are likely to become an increasingly dominant force of change in high Arctic wildlife communities. A proper understanding of not only population dynamics and phenotypic selection processes, but also the trophic interactions and indirect pathways for environmental effects, may thus be key to understand species’ adaptations and resilience to ECEs and rapid climate change.

## Methods

### Study system

The barnacle goose is a migratory, long-lived species, relying, in part, on resources accumulated during migration for subsequent reproduction^62,63^. Thus, individuals’ body condition is important for successful reproduction^61^, growth of offspring^32^, and post-fledging survival^61^. Svalbard barnacle geese overwinter in the UK (54°58′ N, 3°30′ W). They depart in March and migrate to Svalbard for breeding, with a stopover in spring along the Norwegian mainland coast. The study population breeds on western Svalbard, close to the research settlement of Ny-Ålesund (78°55′ N, 11°56′ E). Individuals arrive in late May and nest on islands in the fjord. Spring conditions determine the timing of egg-laying, hatching occurs from late June when families leave the islands to forage. Offspring fledge at the end of August and all individuals return south in September.

### Data collection

Over the study period for selection analyses (1991–1999), females were caught during the breeding season, more specifically during the moulting period when parents raise their offspring, and ringed with colour and metal identification rings. Recapture data were based on daily observations of ringed individuals around Ny-Ålesund. We assessed reproduction based on observations of adults with fledged offspring at the beginning of August. The number of recruits the following year was based on parentage data where families were monitored after capture (and ring marking) to attribute parent–offspring relationships^64^.

Individual body mass and tarsus length were measured during catches in the moulting period, when geese are rearing their chicks. The total tarsus length was measured according to^65^. Body mass reflects the reserves an individual has accumulated^66^, while tarsus length is considered the most reliable indicator of structural size in geese^67^. We performed a linear regression of body mass on tarsus length, and the residuals from this regression were used as an index of individual- and year-specific goose body condition. In another population of barnacle geese, heritability (h^2^) of body mass and tarsus length have been estimated as 0.48 and 1.28, respectively^48,49^.

Annual estimates of barnacle goose population size at Ny-Ålesund were obtained from the results of an integrated population model^40^. The percentage occupied dens was used as a proxy of abundance Arctic foxes, based on annual records of known den sites around Ny‐Ålesund with pup production during summer^28,68^. Arctic foxes are the main predator of juvenile (and, occasionally, adult) geese and thus key in top-down trophic interactions in this system^28,30,32^. Data on reindeer abundance on Brøggerhalvøya from total population counts on snow mobiles in late winter (April) were obtained from^29,69,70^. The annual total amount of rain (i.e., proxy of rain-on-snow) in winter (November–April) was calculated from annual time series of total precipitation data from the weather station at Ny-Ålesund (https://seklima.met.no/)^31^.

### Modelling goose demographic rates

We modelled the effects of variation in barnacle goose body condition or body mass and breeding population size on demographic rates. Fledgling (first year) and adult (two years and older) survival rates were estimated from individual-based mark-recapture data within a Cormack-Jolly-Seber (‘CJS’) framework using the RMark interface^71^ for program MARK^72^. We modelled detection probabilities with a fixed year effect. Generalised linear mixed-effects models (GLMMs) were fitted to the reproductive data, which were modelled as two response parameters: reproductive rate and fledged brood size. Annual reproduction rate, describing whether or not a female had at least one fledgling (0 or 1), was fitted as a binomial response. Fledged brood size describes the number of fledglings per mother and was fitted as a Poisson response. We included observations from 2-year-olds (i.e., age of sexual maturity) onwards in the reproductive models and only successfully reproducing individuals in the model of fledged brood size. GLMMs of the reproductive rate and fledged brood size were fitted with individual bird ID (‘id’) and year random effects using the package *lme4* in R^73^. We performed a model selection including body condition (or body mass), goose population size and the interaction between them as covariates to determine the most parsimonious model of each demographic rate. Akaike’s information criterion adjusted for small sample sizes (AICc) was used to identify the best‐fitting model, where competing models with a difference of AICc score of less than 2 were considered to have the same support ^74^.

### Modelling eco-evolutionary dynamics

Female barnacle goose fitness was defined as the female’s annual contribution in terms of individuals to the following breeding season,$W=I+B/2$, where *I* is survival, *B* is the number of recruits of both sexes to a future breeding season^75^. *I* takes a value of 1 if the female survived to the following breeding season and 0 otherwise, without distinguishing between permanent emigration and mortality. *B*/2 is the expected number of female recruits, assuming an equal sex ratio. This approach includes offspring’s first-year survival in the mother’s fitness and assumes that the number of males is not limiting mating.

When modelling density-dependent selection in a stochastic environment, the relevant measure of fitness is the mean of the fluctuating Malthusian fitness^75–77^:

1 $$m\left(x,N\right)=\mathrm{ln}E\left(W|x,N,\varepsilon \right)=s\left(x\right)- \gamma \left(x\right)N$$

where $\mathrm{ln}EW$ is the expected fitness for individuals with phenotype *x* at population size *N* in environment *ε*, $s\left(x\right)={r}_{0}\left(x\right)-{\sigma }_{e}^{2}/2$ is the long-run growth rate given by ${r}_{0}\left(x\right)$, the deterministic growth rate at small population sizes of phenotype *x*, and ${\sigma }_{e}^{2}$ the environmental variance, and $\gamma \left(x\right)$ is the strength of density dependence of individuals with phenotype *x* to a change in *N*. For a population with mean phenotype $\overline{x }$, the expected Malthusian fitness is $\widetilde{m}\left(\overline{x },N\right)=\overline{s }\left(\overline{x }\right)-\overline{\gamma }\left(\overline{x }\right)N$, where $\overline{s }\left(\overline{x }\right)$ is the mean long-run growth rate in absence of density regulation and $\overline{\gamma }\left(\overline{x }\right)$ is the average strength of density dependence for the mean phenotype in the population^76,77^.

Here, we modelled density-dependent selection on body condition of Svalbard barnacle geese using annual estimates of mark-recapture, reproduction and population size, and pedigree data (see *Data collection)*. The deterministic growth rate at small population sizes ${r}_{0}\left(x\right)$ was defined as a second-degree approximation ${r}_{0}\left(x\right)={\beta }_{1}+{\beta }_{2}x+{\beta }_{3}{x}^{2}$, where ${\beta }_{1}$ is the average deterministic growth rate, ${\beta }_{2}$ is the strength of directional selection, and ${\beta }_{3}$ is the strength of stabilizing selection. The strength of density dependence $\gamma \left(x\right)$ was defined as $\gamma \left(x\right)={e}^{{\alpha }_{1}+{\alpha }_{2}x}$, ensuring strict negative density dependence, where ${\alpha }_{1}$ is the strength of density dependence, and ${\alpha }_{2}$ is the strength of density-dependent selection. Combining these definitions and following Eq. 1, we get the following general linear mixed effect model:

2 $$\mathrm{ln}{\text{E}}\left(2W|x,N,\varepsilon \right)={\beta }_{1} ^{\prime}+{\beta }_{2}x+{\beta }_{3}{x}^{2}-N{e}^{{\alpha }_{1}+{\alpha }_{2}x}+\varepsilon$$

where $2W=2I+B$ is double the female fitness to ensure a Poisson distribution with log link function, $\beta _{1} ^{\prime } = \beta _{1} + {\text{ln}}\left( 2 \right)$, with $\mathrm{ln}\left(2\right)$ added because we fitted the model to 2*W* instead of *W*, and $\varepsilon \sim \mathcal{N}\left(0,{\sigma }_{e}^{2}\right)$ a random year effect drawn from a normal distribution with mean 0 and variance ${\sigma }_{e}^{2}$.

There have been substantial changes in population size since this population colonised the breeding grounds at Kongsfjorden, western Svalbard in the late 1980s. However, there was a marked shift in population sizes between 1995 and 1996. Mean population size in the first part of the selection study period (1991–1995) was 520, which increased to 620 in the second period (1996–1999), with relatively little within-period temporal variation (Fig. 1d). In a second modelling step, we modelled density-dependent selection implicitly by splitting the time series in a period of low density (1991–1995) and high density (1996–1999). For each period we then applied the following general linear mixed effect model:

3 $${\text{lnE}}\left( {2W|x,N,\varepsilon } \right) = b_{1} ^{\prime } + b_{2} x + b_{3} x^{2} + \varepsilon$$

where $b_{1} ^{\prime } = b_{1} + {\text{ln}}\left( 2 \right)$, where $\mathrm{ln}\left(2\right)$ is, according to Sæther et al. (2016, Proc Ropy Soc B), added because we fitted the model to 2*W* instead of *W*, and $\varepsilon \sim \mathcal{N}\left(0,{\sigma }_{e}^{2}\right)$ a random year effect drawn from a normal distribution with mean 0 and variance ${\sigma }_{e}^{2}$. Note that the notation of the selection coefficients has changed in Eq. 3 (to $b$) to distinguish them from the selection coefficients in the density-dependent model of Eq. 2 (i.e., $\beta$).

Estimation was done using a Bayesian implementation of Template Model Builder v. 1.9.3^78^ in Stan via the R package tmbstan v. 1.0.9^79^. Traits were normalized (mean = 0, sd = 1) prior to analyses. Priors for all parameters were uninformative (Stan’s default; flat prior from $-\infty$ to $\infty$), except for ${\alpha }_{1}$ and ${\alpha }_{2}$ which were weakly informative to improve convergence (normal distribution with $\mu =-1$ and $\sigma =2$). Using the no-U-turn sampler (NUTS) Markov chain Monte Carlo (MCMC) algorithm^79^, we ran four independent chains with different starting values for 30,000 iterations, with a burn-in of 20,000 iterations, and thinning every 10th observation, resulting in 4000 posterior samples. We used the rank-based convergence diagnostic $\widehat{R}$ (threshold $\widehat{R}$ ≤ 1.01, following^80^) and the effective sample size (threshold ${n}_{\text{eff}}$ ≥ 400, following^80^) to evaluate chain convergence, and the number of post-burn-in divergences to evaluate model bias^81^. Analyses were performed in R version 4.3.1^82^.

### Supplementary Information

Supplementary Information.

## Data Availability

Data supporting the results are archived in the following Dryad data repository: DOI: 10.5061/dryad.hhmgqnknq.
