# Author Correction: Extreme events, trophic chain reactions, and shifts in phenotypic selection

**DOI:** 10.1038/s41598-023-43603-y

**Published:** 2023-09-29

**Authors:** Kate Layton-Matthews, Stefan J. G. Vriend, Vidar Grøtan, Maarten J. J. E. Loonen, Bernt-Erik Sæther, Eva Fuglei, Brage Bremset Hansen

**Affiliations:** 1 Department of Biology, Centre for Biodiversity Dynamics, NTNU, Trondheim, Norway; 2 Norwegian Institute for Nature Research, NINA, Tromsø, Norway; 3 Department of Animal Ecology, Netherlands Institute of Ecology (NIOO‐KNAW), Wageningen, The Netherlands; 4 Arctic Centre, University of Groningen, Groningen, The Netherlands; 5 Norwegian Polar Institute, Tromsø, Norway; 6 Department of Terrestrial Ecology, Norwegian Institute for Nature Research, NINA, Trondheim, Norway

Correction to: *Scientific Reports* 10.1038/s41598-023-41940-6, published online 13 September 2023

In the original version of this Article, the Data availability section was incorrect,

“On acceptance, the data used in this study would be archived in Dryad repository and the data DOI included in the article.”

now reads:

“Data supporting the results are archived in the following Dryad data repository: DOI: https://doi.org/10.5061/dryad.hhmgqnknq.”

The original Article has been corrected.

