# Supplementary material for: Extreme events, trophic chain reactions, and shifts in phenotypic selection
Source: Sci Rep. 2023 Sep 13;13:15181. doi: 10.1038/s41598-023-41940-6 (PMC10499831; doi:10.1038/s41598-023-41940-6)
Supplement: Supplementary file 1 — Supplementary Information. [file 41598_2023_41940_MOESM1_ESM.docx]

**Supplementary information**

**Appendix S1.** Model selection of demographic rate-morphological trait relationships (body mass and body condition) based on 1991-2017 data.

For the mark-recapture models of survival, body mass (bm) and body condition (bc) were included as an individual- and time-varying covariates, in the case of missing values – which were not allowed in the mark-recapture framework – for an individual with at least one measurement, the mean trait value was used, for individuals where no measurements were taken, the age class average was used. While this approach should not bias estimates, it likely minimises the variance to some extent (see Chapter 11.6, Cooch, 2008). A minimum of one measure of body condition (i.e., both body mass and tarsus length) was available for 1538 of the 1669 individuals in the mark-recapture dataset and a total of 2721 measures of body condition over the study period (1990-2017).

The reproductive models of reproductive rate and fledged brood size, which were fitted as generalised linear mixed effects models (GLMMs), where year and unique bird ID (id) were included as random effects. The tables below show the linear predictor for each candidate model, number of parameters (k), AIC adjusted for small sample size (AIC_c_), difference in AIC_c_ from a model and the lowest AIC_c_ model (ΔAIC_c_) and the model weight. Age class is a factor indicating whether an individual is a fledging (first year of life) or older (adult), N to population size of barnacle geese and intercept to the intercept model.

**Appendix S1.1.** Model selection tables for demographic rates including body condition (residuals from a linear regression of individual tarsus length against individual body mass as the response, number of measurements = 2752) as a covariate.

**Table S1.1.1.** Age class-specific survival.

| **Model** | **k** | **AICc** | **ΔAICc** | **Weight** |
| --- | --- | --- | --- | --- |
| ageclass + bc + ageclass: bc + N + N: bc + N:ageclass + N:ageclass:bc | 35 | 16663.42 | 0.00 | 0.75 |
| ageclass + bc + ageclass:bc + N + N:bc | 33 | 16666.60 | 3.18 | 0.15 |
| ageclass + bc + ageclass:bc + N + N:bc + N:ageclass | 34 | 16668.45 | 5.03 | 0.06 |
| ageclass + bc + ageclass:bc + N + N:ageclass | 32 | 16669.94 | 6.53 | 0.03 |
| ageclass + bc + ageclass:bc + N | 33 | 16672.05 | 8.64 | 0.01 |
| ageclass + bc + ageclass:bc | 31 | 16676.59 | 13.17 | 0.00 |

**Table S1.1.2.** Reproductive rate.

| **Model** | **df** | **AICc** | **ΔAICc** | **Weight** |
| --- | --- | --- | --- | --- |
| bc + N + bc:N | 6 | 1433.80 | 0.00 | 0.76 |
| bc | 4 | 1438.70 | 4.89 | 0.07 |
| intercept | 3 | 1438.80 | 5.03 | 0.06 |
| bc + N | 5 | 1438.90 | 5.06 | 0.06 |
| N | 4 | 1439.00 | 5.17 | 0.06 |

**Table S1.1.3.** Fledged brood size.

| **Model** | **k** | **AICc** | **ΔAICc** | **Weight** |
| --- | --- | --- | --- | --- |
| bc | 3 | 1215.60 | 0.00 | 0.35 |
| intercept | 2 | 1215.80 | 0.17 | 0.32 |
| bc + N | 4 | 1217.50 | 1.91 | 0.13 |
| pop | 3 | 1217.60 | 2.04 | 0.13 |
| bc + N + bc:N | 5 | 1218.50 | 2.91 | 0.08 |

**Appendix S1.2.** Model selection tables for demographic rates including body mass (number of measurements = 2814) as an individual- and time-varying covariate.

**Table S1.2.1.** Age class-specific survival.

| **Model** | **k** | **AICc** | **ΔAICc** | **Weight** |
| --- | --- | --- | --- | --- |
| ageclass + bm + ageclass:bm + N + N:bm + N:ageclass + N:ageclass:bm | 35 | 16674.44 | 0 | 0.98 |
| ageclass + bm + ageclass:bm + N | 32 | 16684.02 | 9.58 | 0.01 |
| ageclass + bm + ageclass:bm + N + N:bm | 33 | 16684.77 | 10.33 | 0.01 |
| ageclass + bm + ageclass:bm + N + N:ageclass | 33 | 16684.95 | 10.51 | 0.01 |
| ageclass + bm + ageclass:bm + N + N:bm + N:ageclass | 34 | 16686.62 | 12.18 | 0 |
| ageclass + bm + ageclass:bm | 31 | 16692.04 | 17.61 | 0 |

**Table S1.2.2.** Reproductive rate.

| **Model** | **k** | **AICc** | **ΔAICc** | **Weight** |
| --- | --- | --- | --- | --- |
| bm + N + bm:N | 6 | 2144.46 | 0.00 | 0.88 |
| bm + N | 5 | 2149.00 | 4.54 | 0.09 |
| bm | 4 | 2151.26 | 6.80 | 0.03 |
| N | 4 | 2158.51 | 14.05 | 0.00 |
| intercept | 3 | 2160.93 | 16.47 | 0.00 |

**Table S1.2.3.** Fledged brood size.

| **Model** | **k** | **AICc** | **ΔAICc** | **Weight** |
| --- | --- | --- | --- | --- |
| intercept | 2 | 2155.01 | 0.00 | 0.46 |
| bm | 3 | 2156.48 | 1.47 | 0.22 |
| N | 3 | 2156.75 | 1.74 | 0.19 |
| bm + N | 4 | 2158.22 | 3.22 | 0.09 |
| bm + N + bm:N | 5 | 2160.24 | 5.24 | 0.03 |

**Appendix S2.** Parameter estimates from analyses of variation in fitness 2*W* as a function of body condition (Table S2.1), body mass (Table S2.2) and tarsus length (Table S2.3). Using the no-U-turn sampler (NUTS) Markov chain Monte Carlo (MCMC) algorithm, we ran four independent chains with different starting values for 30,000 iterations, with a burn-in of 20,000 iterations, and thinning every 10th observation, resulting in 4000 posterior samples. The rank-based convergence diagnostic $\hat{R}$, and the effective sample size $n_{\text{eff}}$ were used to assess chain convergence. Estimates are presented as posterior median and 95% credible interval (95% CrI). $\beta_{1}=\beta_{1}^{'}-\ln\left( 2 \right)$ and $b_{1}=b_{1}^{'}-\ln\left( 2 \right)$ are the corrected intercepts because of fitting the model to 2*W* instead of *W*. $P_{0}$ is the proportion of posterior samples overlapping with zero. Period 1 corresponds to years 1991-1995 and Period 2 to 1996-1999, and *N* refers to population size.

**Table S2.1**. Parameter estimates from analyses of variation in fitness 2*W* as a function of body condition *x*.

| **Parameter** | **Median** | **95% CrI** | $\hat{R}$ | $n_{\text{eff}}$ | $P_{0}$ |
| --- | --- | --- | --- | --- | --- |
| **Model:** $\ln\text{E}\left( 2W \vert x,N,\varepsilon\right)=\beta_{1}^{'}+\beta_{2}x+\beta_{3}x^{2}-Ne^{\alpha_{1}+\alpha_{2}x}+\varepsilon$ | | | | | |
| $\beta_{1}$ | 0.191 | 0.035 – 0.333 | 1.001 | 1881 | 0.013 |
| $\beta_{2}$ | 0.136 | 0.087 – 0.183 | 1.000 | 2044 | 0.000 |
| $\beta_{3}$ | -0.037 | -0.069 – -0.009 | 0.999 | 2061 | 0.004 |
| $\alpha_{1}$ | -2.799 | -5.187 – -1.626 | 0.999 | 1933 | 0.000 |
| $\alpha_{2}$ | -0.531 | -1.442 – -0.025 | 1.000 | 2001 | 0.022 |
| $\ln\left( \sigma\right)$ | -1.632 | -2.201 – -0.970 | 1.000 | 1783 | 0.000 |
| **Model:** $\ln\text{E}\left( 2W \vert x,N,\varepsilon\right)=b_{1}^{'}+b_{2}x+b_{3}x^{2}+\varepsilon$ | | | | | |
| **Period 1 (low N)** | | | | | |
| $b_{1}$ | 0.269 | -0.140 – 0.676 | 1.002 | 1726 | 0.058 |
| $b_{2}$ | 0.068 | 0.007 – 0.133 | 1.000 | 1785 | 0.015 |
| $b_{3}$ | -0.044 | -0.085 – -0.007 | 1.002 | 1823 | 0.012 |
| $\ln\left( \sigma\right)$ | -1.205 | -1.979 – 0.053 | 1.000 | 3725 | 0.025 |
| **Period 2 (high N)** | | | | | |
| $b_{1}$ | 0.086 | -0.209 – 0.376 | 1.000 | 1058 | 0.182 |
| $b_{2}$ | 0.207 | 0.131 – 0.278 | 1.003 | 1819 | 0.000 |
| $b_{3}$ | -0.021 | -0.065 – 0.022 | 1.000 | 2088 | 0.175 |
| $\ln\left( \sigma\right)$ | -1.809 | -3.548 – -0.216 | 1.000 | 1567 | 0.020 |

**Table S2.2**. Parameter estimates from analyses of variation in fitness 2*W* as a function of body mass *x*.

| **Parameter** | **Median** | **95% CrI** | $\hat{R}$ | $n_{\text{eff}}$ | $P_{0}$ |
| --- | --- | --- | --- | --- | --- |
| **Model:** $\ln\text{E}\left( 2W \vert x,N,\varepsilon\right)=\beta_{1}^{'}+\beta_{2}x+\beta_{3}x^{2}-Ne^{\alpha_{1}+\alpha_{2}x}+\varepsilon$ | | | | | |
| $\beta_{1}$ | 0.211 | 0.026 – 0.376 | 1.001 | 1872 | 0.015 |
| $\beta_{2}$ | 0.141 | 0.095 – 0.188 | 1.003 | 2022 | 0.000 |
| $\beta_{3}$ | -0.036 | -0.065 – -0.008 | 1.000 | 4075 | 0.004 |
| $\alpha_{1}$ | -2.704 | -4.853 – -1.567 | 1.003 | 2101 | 0.000 |
| $\alpha_{2}$ | -0.581 | -1.374 – -0.105 | 1.001 | 2215 | 0.011 |
| $\ln\left( \sigma\right)$ | -1.477 | -2.052 – -0.760 | 1.000 | 1850 | 0.000 |
| **Model:** $\ln\text{E}\left( 2W \vert x,N,\varepsilon\right)=b_{1}^{'}+b_{2}x+b_{3}x^{2}+\varepsilon$ | | | | | |
| **Period 1 (low N)** | | | | | |
| $b_{1}$ | 0.283 | -0.097 – 0.642 | 0.999 | 1696 | 0.048 |
| $b_{2}$ | 0.066 | 0.006 – 0.128 | 0.999 | 2148 | 0.017 |
| $b_{3}$ | -0.039 | -0.081 – -0.004 | 0.999 | 2114 | 0.017 |
| $\ln\left( \sigma\right)$ | -1.196 | -2.018 – -0.072 | 0.999 | 1649 | 0.022 |
| **Period 2 (high N)** | | | | | |
| $b_{1}$ | 0.145 | -0.323 – 0.634 | 1.001 | 861 | 0.179 |
| $b_{2}$ | 0.224 | 0.159 – 0.292 | 0.999 | 2035 | 0.000 |
| $b_{3}$ | -0.026 | -0.070 – 0.018 | 1.000 | 2034 | 0.129 |
| $\ln\left( \sigma\right)$ | -1.331 | -2.281 – 0.291 | 1.001 | 1403 | 0.054 |

**Table S2.3**. Parameter estimates from analyses of variation in fitness 2*W* as a function of tarsus length *x*.

| **Parameter** | **Median** | **95% CrI** | $\hat{R}$ | $n_{\text{eff}}$ | $P_{0}$ |
| --- | --- | --- | --- | --- | --- |
| **Model:** $\ln\text{E}\left( 2W \vert x,N,\varepsilon\right)=\beta_{1}^{'}+\beta_{2}x+\beta_{3}x^{2}-Ne^{\alpha_{1}+\alpha_{2}x}+\varepsilon$ | | | | | |
| $\beta_{1}$ | 0.189 | 0.014 – 0.355 | 1.002 | 1870 | 0.023 |
| $\beta_{2}$ | 0.039 | -0.010 – 0.086 | 1.001 | 1931 | 0.053 |
| $\beta_{3}$ | -0.014 | -0.044 – 0.014 | 1.003 | 1927 | 0.172 |
| $\alpha_{1}$ | -3.037 | -5.969 – -1.664 | 1.005 | 1754 | 0.000 |
| $\alpha_{2}$ | -0.331 | -1.047 – 1.100 | 1.002 | 1523 | 0.197 |
| $\ln\left( \sigma\right)$ | -1.497 | -2.065 – -0.800 | 1.000 | 1878 | 0.000 |
| **Model:** $\ln\text{E}\left( 2W \vert x,N,\varepsilon\right)=b_{1}^{'}+b_{2}x+b_{3}x^{2}+\varepsilon$ | | | | | |
| **Period 1 (low N)** | | | | | |
| $b_{1}$ | 0.231 | -0.205 – 0.648 | 1.001 | 1233 | 0.099 |
| $b_{2}$ | -0.005 | -0.067 – 0.058 | 1.000 | 1983 | 0.438 |
| $b_{3}$ | -0.006 | -0.040 – 0.025 | 1.002 | 2058 | 0.357 |
| $\ln\left( \sigma\right)$ | -1.121 | -1.851 – 0.134 | 1.000 | 1655 | 0.134 |
| **Period 2 (high N)** | | | | | |
| $b_{1}$ | 0.115 | -0.300 – 0.599 | 1.000 | 1410 | 0.203 |
| $b_{2}$ | 0.088 | 0.021 – 0.161 | 1.000 | 1908 | 0.004 |
| $b_{3}$ | -0.017 | -0.062 – 0.025 | 1.000 | 2058 | 0.221 |
| $\ln\left( \sigma\right)$ | -1.393 | -2.492 – 0.189 | 1.000 | 1867 | 0.046 |


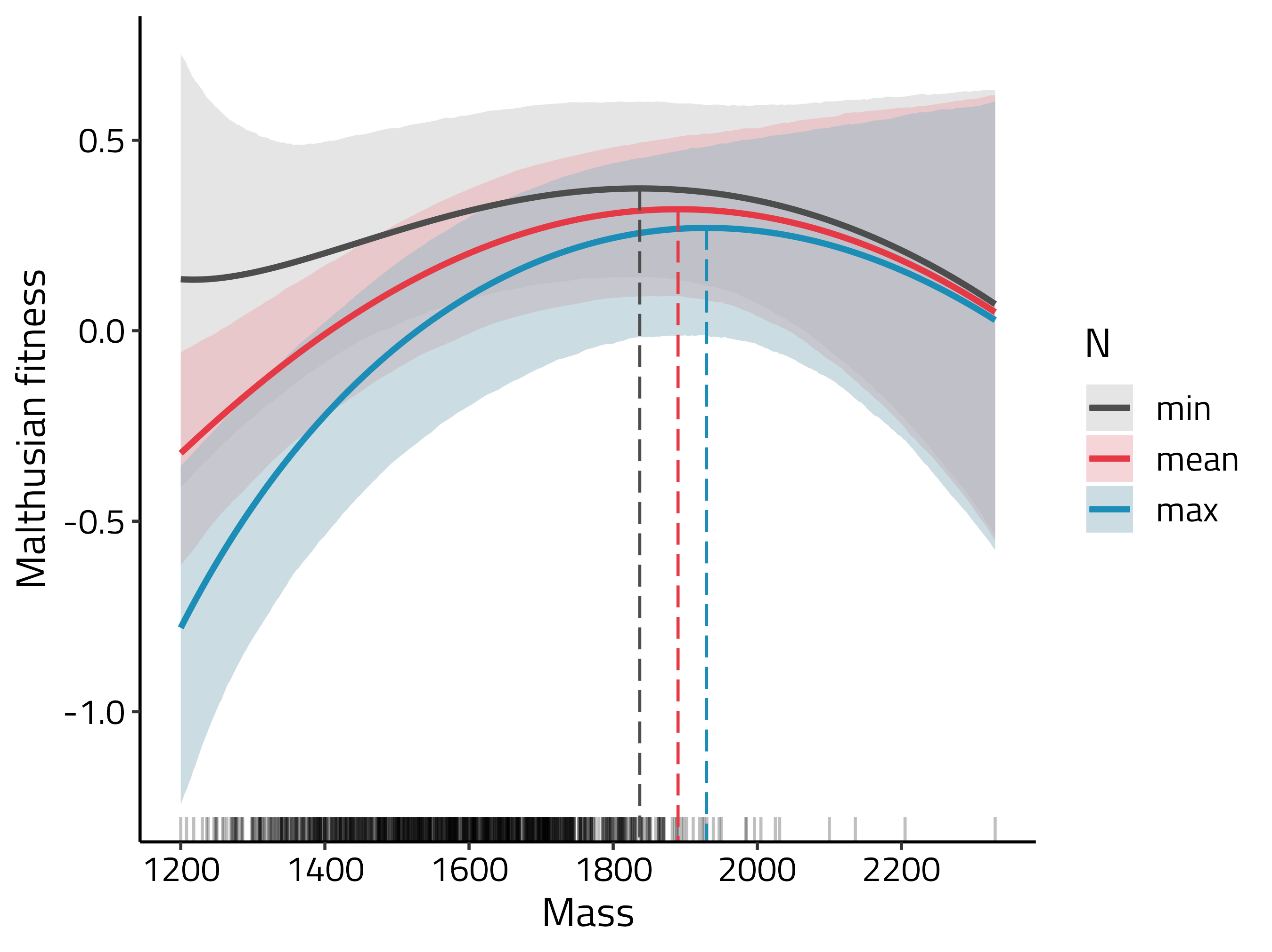


**Figure S2.1.** Malthusian fitness for values of body mass at the minimum (*N* = 566), mean (800) and maximum (1071) population size. Thick lines represent posterior means. Ribbons represent 95% credible intervals. Dashed lines refer to mass values maximizing fitness.


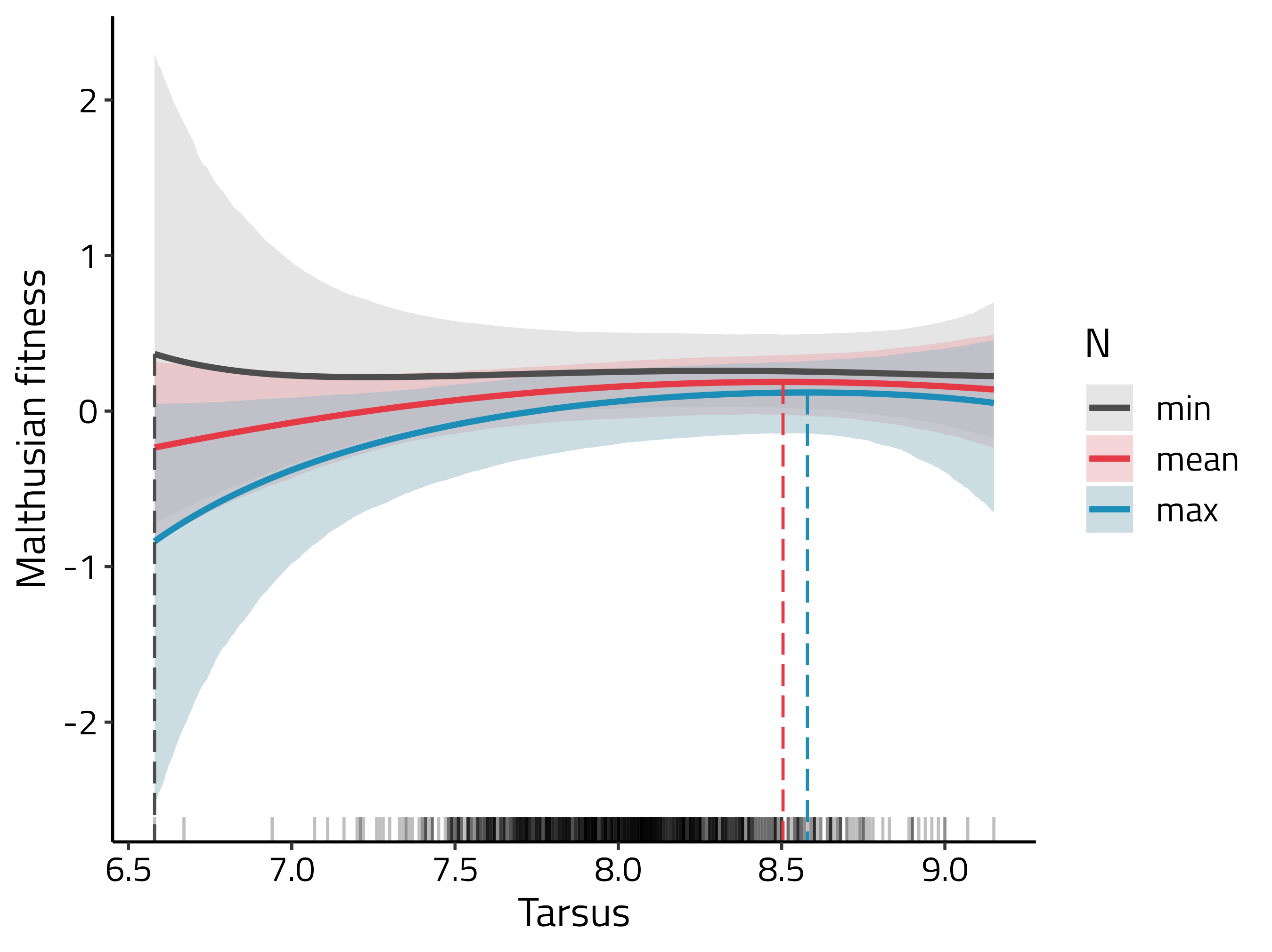


**Figure S2.2.** Malthusian fitness for values of tarsus length at the minimum (*N* = 566), mean (800) and maximum (1071) population size. Thick lines represent posterior means. Ribbons represent 95% credible intervals. Dashed lines refer to tarsus values maximizing fitness.

**
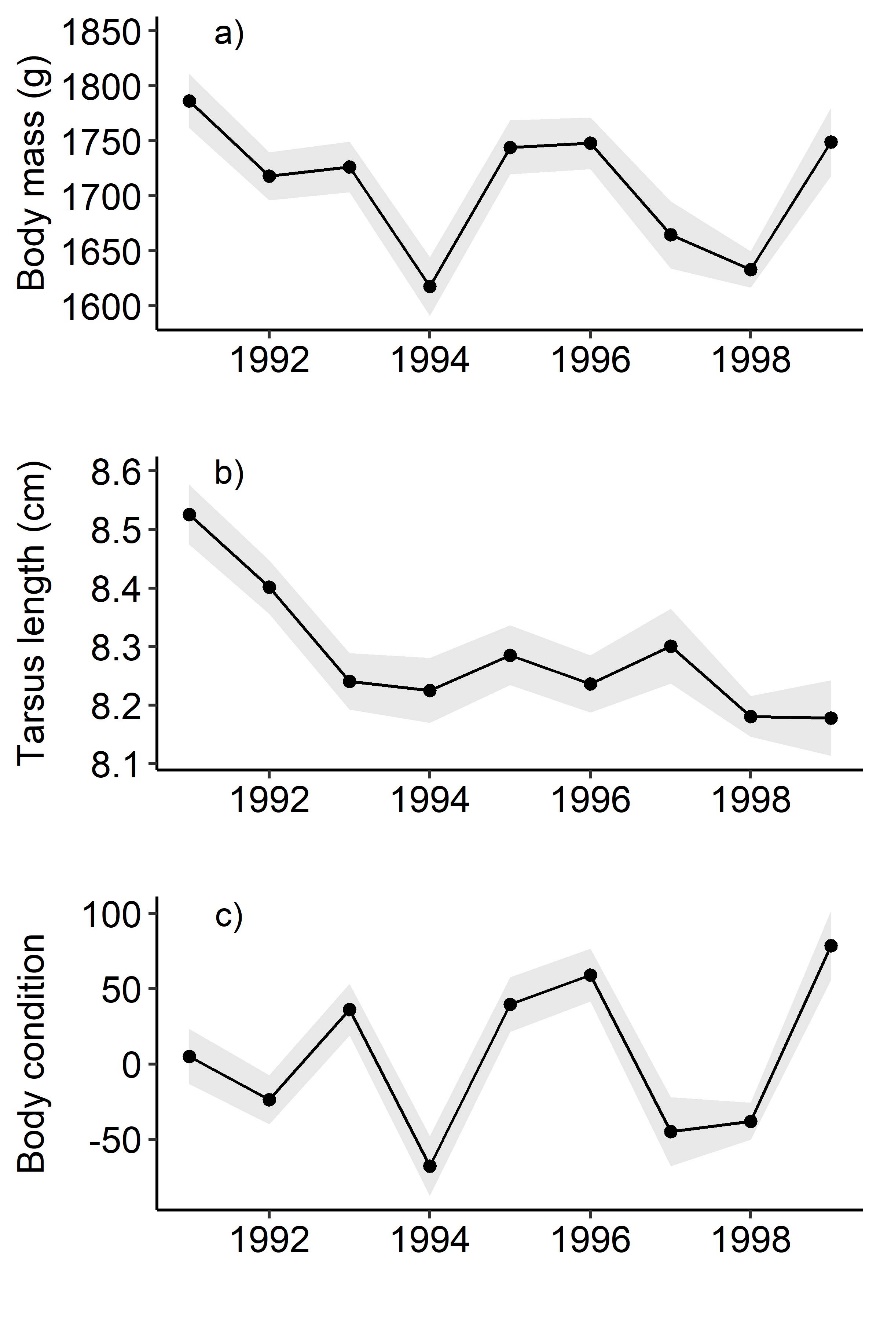
Appendix S3.** Model predictions of annual barnacle goose phenotypic traits; body mass, tarsus length and body condition.

**Fig. S3.1** Model predictions (95% confidence intervals are represented by grey shading) of annual a) body mass, b) tarsus length (proxy for body size) and c) body condition of adult barnacle geese from 1991-1999 (values are scaled to mean = 0 and 1 standard deviation variance), where body condition is measured as the residual of a linear model where individual body mass is regressed against tarsus length. Traits were modelled with a linear model with year (1991-1999).
